# “Knowing It Before Blocking It,” the ABCD of the Peripheral Nerves: Part A (Nerve Anatomy and Physiology)

**DOI:** 10.7759/cureus.41771

**Published:** 2023-07-12

**Authors:** Kartik Sonawane, Hrudini Dixit, Aparna Jayaraj, Navya Thota, Chelliah Sekar

**Affiliations:** 1 Anesthesiology, Ganga Medical Centre and Hospitals, Pvt. Ltd., Coimbatore, IND; 2 Anesthesiology, Sir H. N. Reliance Foundation Hospital and Research Centre, Mumbai, IND; 3 Anesthesiology, Ganga Medical Centre and Hospitals Pvt. Ltd., Coimbatore, IND

**Keywords:** nerve fibers, nerve impulse, nerve connective tissues, regional anesthesia, nerve physiology, nerve anatomy

## Abstract

Regional anesthesia (RA) is an interplay between the local anesthetic (LA) solution and the neural structures, resulting in nerve conduction blockade. For that, it is necessary to understand which hurdles the LA has to overcome and which components of the nerves are involved. Background knowledge of the neural and non-neural components of the nerve helps locate the safest area for LA deposition. In addition, knowledge of nerve physiology and the conduction process helps to understand the patterns of block onset, involved fibers, and block regression. Neural connective tissue protects the nerve on the one hand and influences the overall effect of the blockade and the occurrence of nerve injuries on the other. The arrangement of the nerve fibers explains the science behind the differential blockage after LA deposition.

This article describes the important aspects of nerve anatomy (nerve formation and composition) and nerve physiology (impulse generation and propagation). It also provides insight into the physiological processes involved when a damaged neural structure leads to potential clinical symptoms. It will help readers sharpen their skills and knowledge to execute safe RA without damaging any vital structures in the nerve.

## Introduction and background

The principle of regional anesthesia/analgesia (RA) is to deposit a local anesthetic (LA) solution around nerves or plexuses to cause conduction blockade. Therefore, it is essential to know the details about target structures (nerve anatomy), the process of impulse conduction (nerve physiology), and LA pharmacology. Many examples support the famous quote, "Knowing is half the battle." The success of general anesthesia (GA) induction and maintenance lies in a thorough knowledge of human physiology. A detailed comprehension of the structures helps a surgeon triumph over the surgery. Similarly, the multifaceted application of modern RA requires shifting the entire focus to learning or acquiring essential background knowledge of anatomy, physiology, and pharmacology relevant to the technique.

RA has evolved from the blind technique (paresthesia elicitation) - to the objective technique (peripheral nerve stimulation) - eventually to a more precise real-time visualization technique (ultrasound guidance). It has improved in terms of safety, reliability, and precision. However, expectations of success have also increased. The power of success lies in the anatomy, the backbone of RA, but one must know it first. Ultrasound-guided RA is an art based on technology-supported science. Therefore, looking into the structures (knowing them in detail) is imperative to understanding the science rather than just looking at the structures in the image. By knowing the details of the structures of interest (like nerves or plexuses) and their relationship to the environment, one can anticipate possible complications or damage and automatically take the utmost precautions when handling them.

This article is part of a comprehensive overview of the essential understanding of peripheral nerves before blocking them. It describes the important aspects of nerve anatomy (nerve formation and composition) and nerve physiology (impulse generation and propagation). We believe this article will help readers determine the safest location for LA deposition to block a specific nerve. It also provides insight into the physiological processes involved when a damaged neural structure leads to potential clinical symptoms. Therefore, this article will help sharpen the skills and knowledge needed to ensure safe RA without complications.

## Review

This narrative overview describes the components and functions of nerve tissue, nerve formation and its composition, and the physiology of nerve impulse generation and transmission. Related literature searches were performed using online platforms (PubMed, Medline, and Embase databases, Cochrane Library, and Google Scholar) using relevant search terms (neurons/nerve anatomy/nervous system/nerve connective tissues/nerve physiology/nerve physiological properties/nerve impulse generation/impulse propagation). Articles published in English were selected, and their reference sections were manually searched for additional information.

An overview of the nervous system

The nervous system plays an important role in controlling body activities through the endocrine system. It provides swift and brief responses to different stimuli. It is a major controlling, regulatory, and communicating system and the center of all mental activities like thought, learning, and memory. It acts through electrical impulses and neurotransmitters to cause muscle contractions or glandular secretions. Its effect is of short duration, measured in seconds, and localized [1]. At the same time, the endocrine system is primarily involved in controlling metabolic processes and is responsible for long-term changes in the body. It is a messenger system comprising feedback loops of the hormones released by the internal glands directly into the circulatory system. It influences growth, development, metabolic activities, energy level, reproduction, and response to injury, stress, and mood. Its effect is long, measured in minutes, hours, or weeks, and more generalized [2].

**Figure 1:**
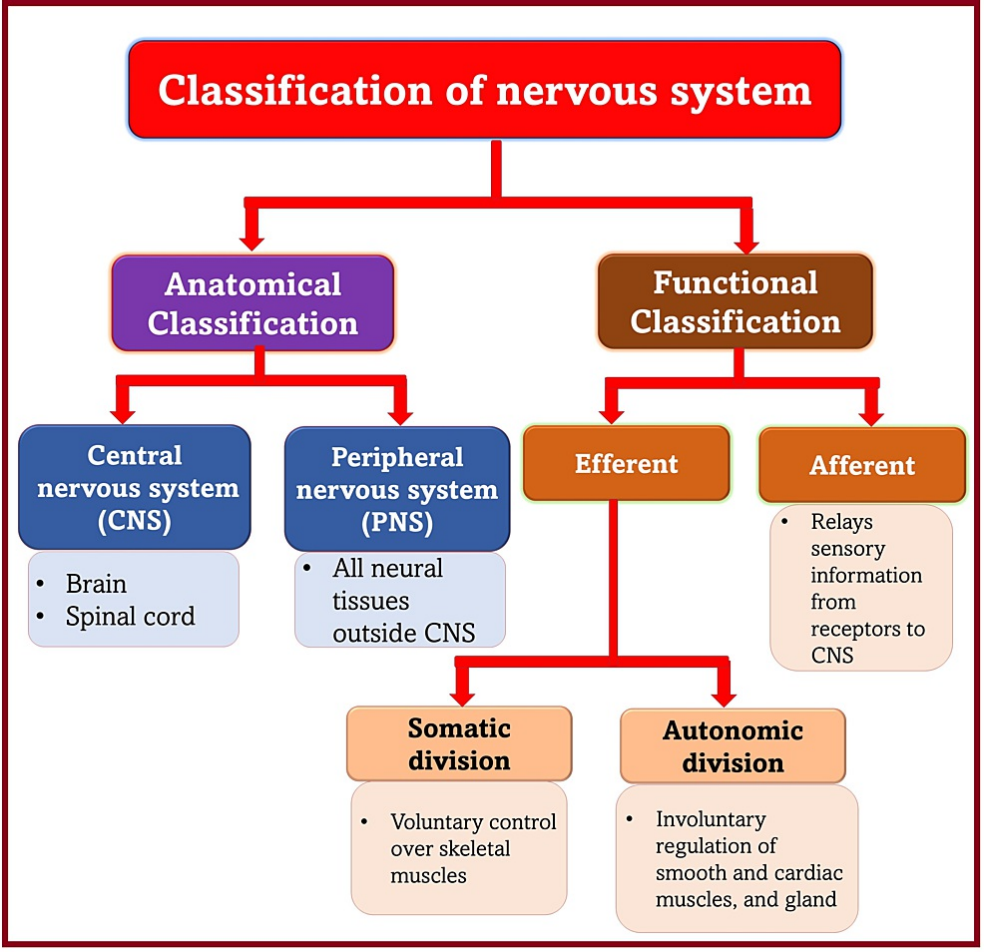
Classification of the nervous system. [3].

This article mainly focuses on the nervous system and its components related to the peripheral nervous system (PNS). The nervous system is classified based on anatomy and function (Figure 1) [3]. It comprises two organs (the brain and spinal cord) consisting of neuronal tissues formed by two types of neural cells: neuroglia (glial cells) and neurons (nerve cells).

Neuroglia (Glial Cells)

**Figure 2:**
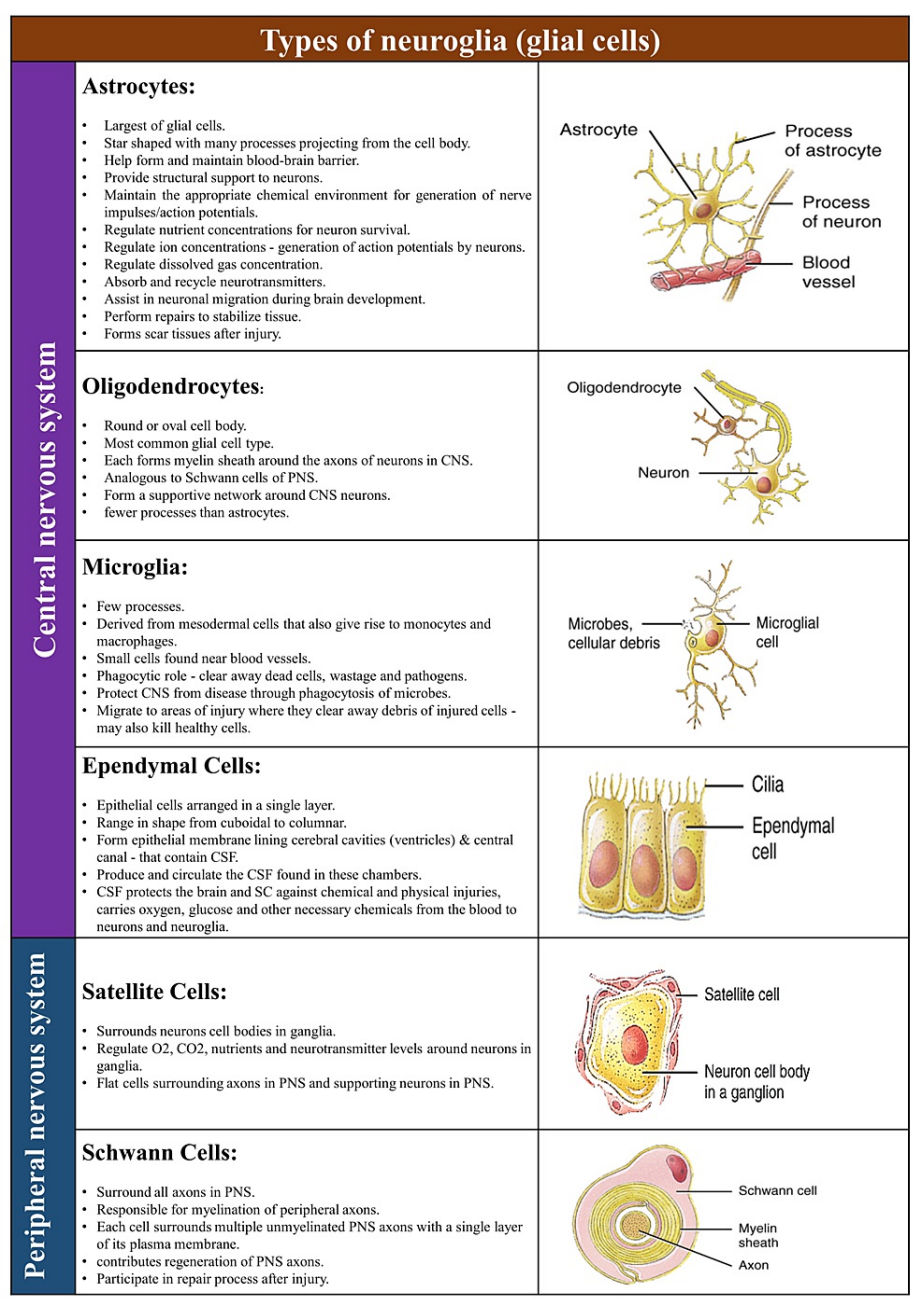
Types of neuroglia. CNS: central nervous system; PNS: peripheral nervous system; CSF: cerebrospinal fluid; SC: spinal cord [3,4]. Source: This figure was created by the first author (KS).

As the name suggests, neuroglia, meaning nerve glue, are nonconductive cells that support, nourish, and protect the neurons (Figure 2) [3,4]. They are smaller and more numerous (10-50 times) than neurons, which occupy about half the cell volume in the central nervous system (CNS) [5]. Unlike neurons, glial cells cannot generate electrical impulses but can divide through mitosis.

Neurons

**Figure 3:**
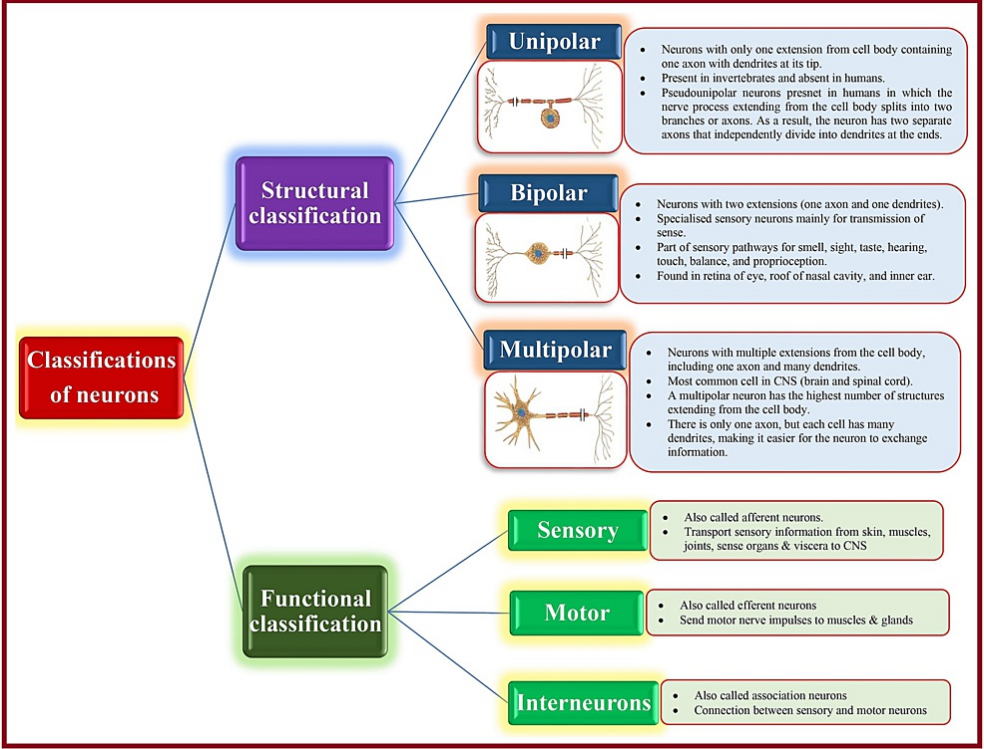
Classification of neurons. CNS: central nervous system [3]. Source: This figure was created by the first author (KS).

In contrast to neuroglia, highly specialized neurons are electrically excitable (the ability to generate action potentials [AP] or impulses in response to stimuli) and amitotic (irreplaceable upon destruction). Neurons are classified based on their structures and functions (Figure 3). Some neurons are named after histologists who described them: Purkinje cells (present in the cerebellum) and Renshaw cells (present in the spinal cord).

Embryologically, neurons are ectodermal derivatives. The CNS components (brain and spinal cord) are derived from the neuroectoderm [6,7]. In comparison, peripheral neurons are derived from neural crest cells. The myelination of CNS axons occurs via oligodendrocytes that are derived from neuroepithelial cells [7]. Whereas myelination of peripheral axons occurs via Schwan cells that are derived from neural crest cells.

The development of the neurons stops before birth, and they remain amitotic. Therefore, dead neurons cannot be replaced. Developmental processes such as nerve migration, maturation, and synaptogenesis are influenced by environmental factors such as sensory stimuli, relationships, hormones, and certain drugs [7,8].

**Figure 4:**
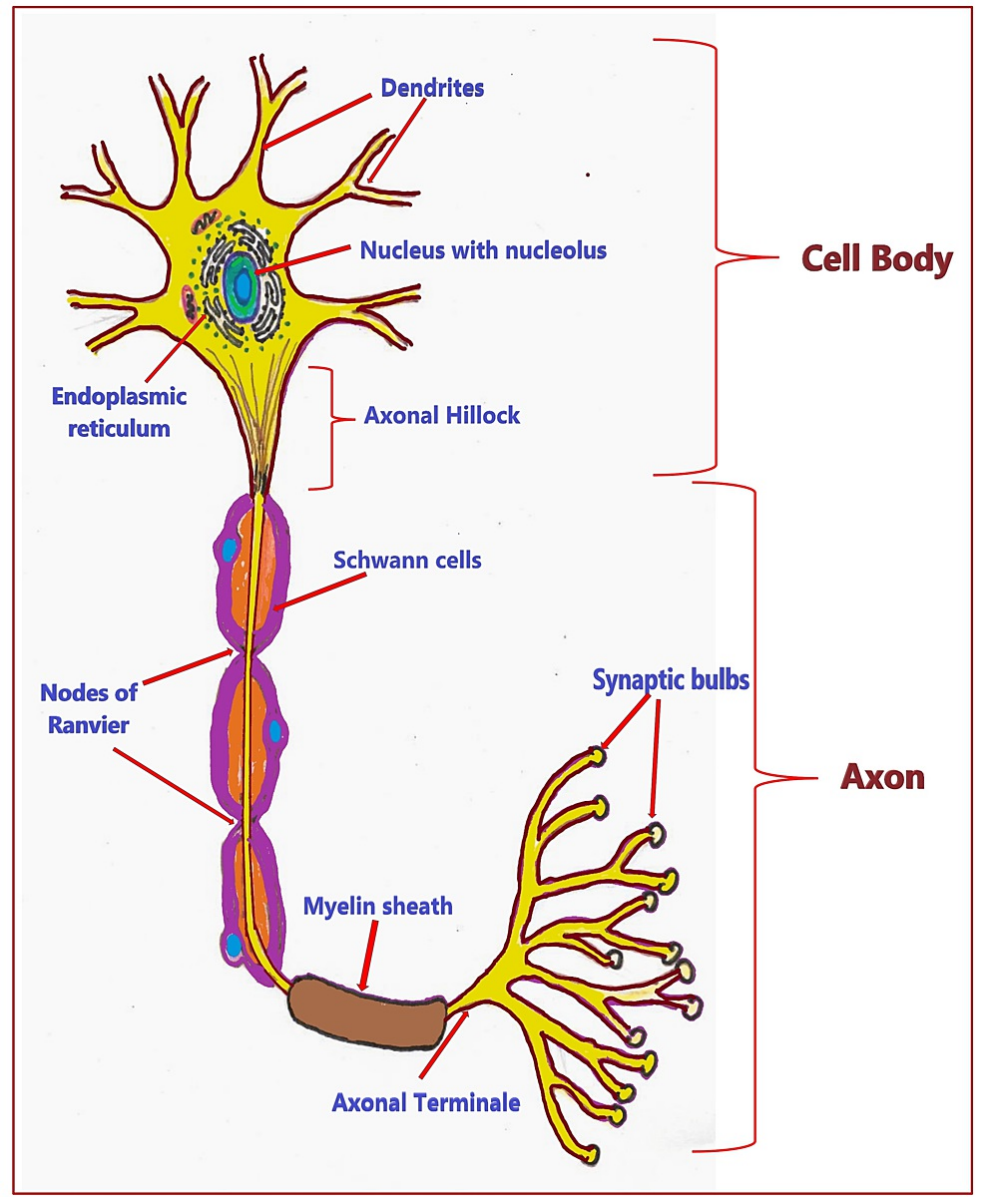
Basic structure of neurons. Source: This figure was created by the first author (KS).

Structure of neurons: The basic structure of neurons (Figure 4) consists of the cell body (or soma/perikaryon) and cell processes, or neurites (dendrite and axon), arising from it. The cell body contains an outer cell membrane, or plasma membrane, enclosing the cytoplasm. The cytoplasm contains various cell organelles having specific roles: the central nucleus with a prominent nucleolus (control center of cellular activities), granules (Nissl bodies), rough endoplasmic reticulum (ER), and free ribosomes (responsible for protein synthesis, required to grow and repair damaged axons), neurofibrils or neurofilaments extend from the dendrites into the axon (responsible for cell shape and support), microtubules (to move material inside the cell), yellowish-brown lipofuscin pigment clumps (harmless aging), Golgi apparatus (transporting, modifying, and packaging proteins and lipids to targeted destinations), and mitochondria (energy generation and storage) [9].

Dendrites: Dendrites (little trees) are also known as the receiving or input portion of the neuron. They have short, tapering, and highly branched surfaces specialized for contact with other neurons. Their cytoplasm contains Nissl bodies and mitochondria. They differ from the axon in several characteristics, including shape (tapering dendrites and constant radius of axons), length (shorter dendrites and longer axons), and function (dendrites usually receive signals while axons transmit them) [10].

Axon: An axon (axis in Greek) is a long, thin, and slender projection of neurons that typically conducts electrical impulses away from the cell body. This primary transmission line of the nervous system transmits information to various neurons, muscles, and glands. Axon diameter may vary from 1 µm to 20 µm and length from 1mm to 1 m [11]. The sciatic nerve axons are the longest and run from the base of the spinal cord to the big toe of the foot. The axon connects to the cell body at a cone-shaped elevation called the axon hillock. A thick and unmyelinated portion of an axon that connects directly to the cell body is known as the initial segment. It is approximately 25 μm long and consists of a specialized protein complex. It functions as the site of AP initiation [12]. The voltage-gated sodium (Na) channels are much denser in the initial segment and axonal hillock than in the remainder of the axon or the adjacent cell body [13]. A plasma membrane surrounding the cytoplasm of the axon (axoplasm) is known as an axolemma. The axoplasm contains mitochondria, microtubules, and neurofibrils but no ER - no protein synthesis [14].

Side branches, or collaterals, arising from the axon end in fine processes called axon terminals. The complex branching pattern structure at axon terminals is also known as axonal arborization. It allows the simultaneous transmission of messages to many target neurons within a single region. The CNS axons typically show complex trees with many branch points. In contrast, the axon of the cerebellar granule cells has a single T-shaped branch node from which two parallel fibers radiate [10]. Axon terminals have swollen tips at the end called synaptic end bulbs, which contain vesicles filled with neurotransmitters. Axons make contact with other neurons, muscle, or gland cells at junctions called synapses. At a synapse, the axon membrane closely adjoins the target cell membrane. Electrical or electrochemical signals are transmitted across the synaptic cleft by special molecular structures. Most impulses arise at the junction of the axon hillock with the initial segment, called the trigger zone [15]. The voltage-gated ion channels within certain areas of the axonal membrane help initiate AP, conduction, and synaptic transmission [10].

Depending on the presence of myelin, axons can be unmyelinated or myelinated in the CNS and PNS [10]. Myelin is a fatty insulating layer formed by Schwann cells in the PNS and oligodendrocytes in the CNS. The sheath of Schwann cells lies outside the myelin sheath, called the neurilemma. Both the neurilemma and myelin sheath constitute a medullary sheath that is interrupted at intervals by the nodes of Ranvier. The nodes of Ranvier, also called myelin sheath gaps, are short, unmyelinated segments of a myelinated axon. At each node of Ranvier, the axon is reduced in diameter [16]. Axons, Schwann cells, and the myelin sheath constitute nerve fiber. Many myelinated and unmyelinated nerve fibers are grouped together to form a peripheral nerve.

Formation of peripheral nerves

**Figure 5:**
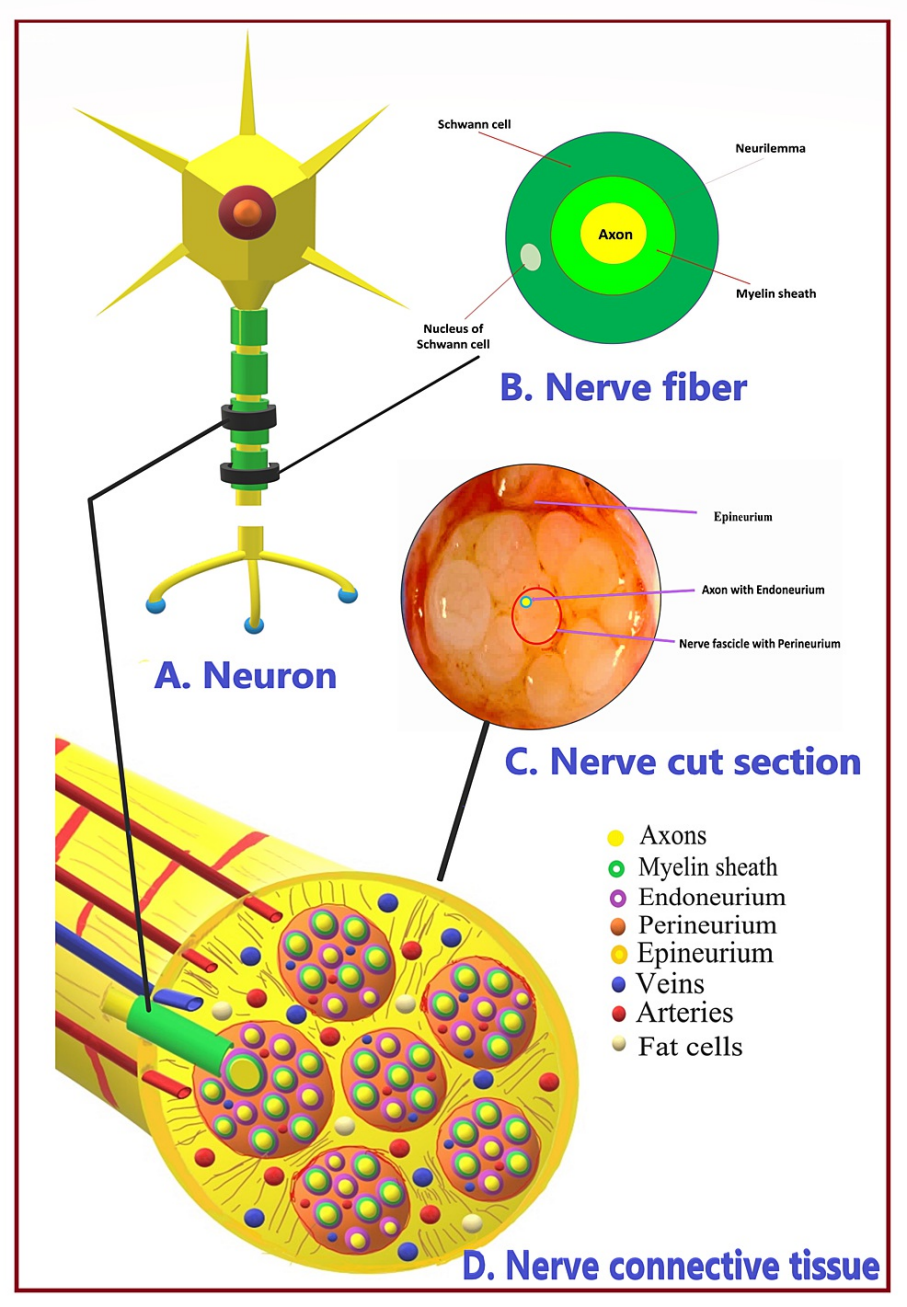
Neural and non-neural components of the peripheral nerve. Source: This figure was created by the first author (KS).

Each nerve comprises nervous tissues consisting of nerve cells (neurons and neuroglia) and supporting connective tissues. Both constitute neuronal and non-neuronal components of the nerve. The neuronal component includes nerve fibers (Figure 5), consisting of the axon, myelin sheath (if present), and Schwann cells. In comparison, the non-neuronal component comprises connective tissues surrounding dendrites and nerve fibers. The amount of neural tissue remains constant, but the non-neuronal component increases from the origin to the distal part of the nerve. The cross-sectional area of a peripheral nerve may consist of up to 70% loose connective tissue [17]. The non-neuronal component provides inherent protection for nerves as they traverse across the joint and fascial planes, thus protecting against compression and stretching [18].

The ratio of non-neural to neural components within the epineurium increases from 1:1 in the proximal plexus to 2:1 in the distal plexus [17]. For instance, the brachial plexus elements below the clavicle have a higher connective tissue-to-neural tissue ratio than proximal trunks and divisions [19]. Similarly, the sciatic nerve at the popliteal fossa contains more non-neural tissue than fascicles in its cross-sectional area [17]. Because of this arrangement, the proximal portion of the nerve appears on ultrasound as a hypoechoic halo surrounded by a thin hyperechoic rim. In contrast, the distal portion of the nerve appears as a small hypoechoic halo surrounded by a thick hyperechoic layer (stippled or honeycomb appearance). When needling and depositing LA perineurally, it is important to know the arrangement of neuronal and non-neuronal parts of the nerves. When a needle inadvertently enters a peripheral nerve, it does not always end up in a fascicle but may lie in connective tissue. The neuronal component of the nerve lies in the nerve fascicle, which must be protected during peripheral nerve blocks to avoid significant nerve injury.

Nerve Fascicle

Each nerve consists of bundles of nerve fibers arranged in groups called nerve fascicles (Figure 5). The nerve can have a single fascicle (monofascicular) or multiple fascicles (multifascicular), like a large nerve. Fascicular bundles divide every few millimeters and anastomose with each other throughout the peripheral nerve [20]. However, within the small set of adjacent bundles, the axons redistribute and remain in approximately the same quadrant of the nerve for several centimeters [21]. This arrangement is of practical concern to surgeons trying to repair severed nerves. Each nerve fiber in the fascicle is surrounded by a delicate tissue matrix known as endoneurium, forming a continuous tube extending from the spinal cord to the synapse level. A robust epithelial sheath, the perineurium, holds all fascicles together that is further supported and encased by loose collagen fibers, the epineurium.

Endoneurium

The endoneurium is also called an endoneurial channel, endoneurial tube, or Henle’s sheath. It is a thin, delicate, and protective layer of connective tissue around the myelin sheath of each myelinated nerve fiber or unmyelinated axon. The endoneurium consists of an inner sleeve of the glycocalyx and a delicate outer meshwork of collagen fibers [22]. Among the various endoneurial cells [23], fibroblasts are the most abundant and are responsible for fiber formation and ground substance production. An endoneurial fluid containing low proteins lies inside the endoneurium. It corresponds to cerebrospinal fluid in the CNS. The endoneurial fluid pressure is higher than that of the surrounding epineurium. This pressure gradient helps minimize endoneurial contamination by external toxic substances [24]. The endoneurial microvasculature and the innermost layer of the perineurium constitute a blood-nerve barrier [25]. It prevents substances from crossing the bloodstream into the endoneurial fluid. If the nerve is injured or irritated, the endoneurial fluid increases, causing nerve edema. Magnetic resonance neurography (MRN) can detect changes in endoneurial fluid volume that suggest nerve injury [26].

Perineurium

The perineurium surrounds each nerve fascicle with a distinct lamellar arrangement of seven to eight concentric layers [27]. It protects and supports the nerve fibers and prevents the passage of macromolecules from the outside into the fascicle. Perineurial cells (mainly epitheloid and myofibroblasts) have properties like contractility, tight and gap junctions, and external laminae. They are metabolically active and play a role in maintaining electrolyte and glucose balance around nerve cells. The cytoplasm of perineurial cells contains small amounts of ER, filaments, and numerous endocytic vesicles. Tight and gap junctions are present between adjacent cells within the same layer of the perineurium and between successive layers [28]. Tight junctions of the inner perineurium and the endoneurial capillaries form a blood-nerve barrier, as mentioned before [29]. The perineurium maintains the intrafascicular pressure and contributes to the barrier effect [30-32]. The pressure exerted on the perineurium is transmitted to the endoneurium and nerve fibers. The increased thickening of the perineurium at nerve branching offers additional protection. Such thickening also helps limit the spread of infections and inflammatory reactions [23]. However, the infection can easily spread across nerve fascicles when the perineurium is not intact. The tough perineurium also acts as a semipermeable barrier to LA [33,34].

Epineurium

The epineurium is the outermost layer of dense, irregular connective tissue that surrounds a peripheral nerve [35], representing between 30% and 75% of the cross-sectional area of a nerve. The proportion of epineurium is higher in larger nerves with increasing numbers of nerve fascicles. However, the epineurium is absent around monofascicular nerves and at nerve endings. The epineurium also projects longitudinal "ondulations" along its trajectory, which provide nerve elasticity [23]. It surrounds multiple nerve fascicles and blood vessels supplying the nerve (vasa nervosa). The epineurial (extrinsic) blood vessels communicate with the perineurial (intrinsic) blood vessels. The epineurial blood vessels and lymphatics run longitudinally and parallel to the nerve fascicles. Injury to the epineurium, however, does not compromise axonal safety to the same extent. In the case of ischemia, the epineural barrier effect can be maintained up to 22 hours postmortem [31]. This barrier effect is lost following nerve lesions and recovers between 2 and 30 days after injury [30].

Paraneurium

**Figure 6:**
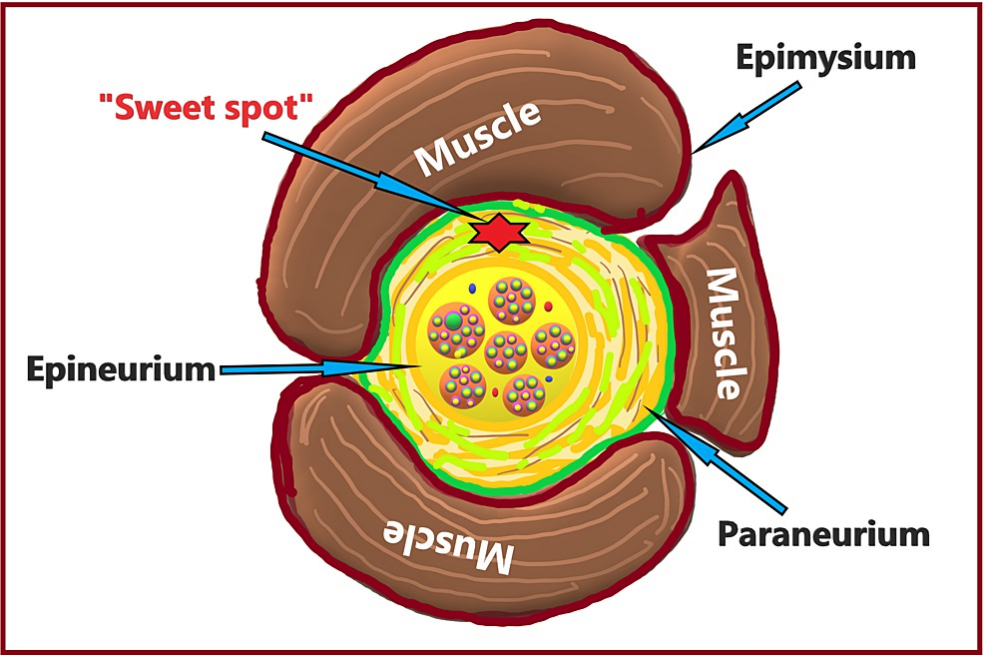
Paraneural sheath or "sweet spot" of the nerve. Source: This figure was created by the first author (KS).

The paraneurium is also known as the paraneural sheath, mesoneurium [36], common epineural sheath [37], conjuctiva nervorum [38], or adventitia [39]. It refers to the loose connective tissue between the epineurium of the peripheral nerve and the epimysium of the surrounding muscles (Figure 6). It facilitates the longitudinal displacement of nerves and avoids compression or stretching of nerves during body movement. The epineurium and paraneurium together protect the nerve from injury by providing insulation. The sciatic nerve sheath is the compact paraneurium (a common epineural sheath) enclosing two nerves (the tibial and common peroneal nerves) with their epineurium. The paraneurium is also referred to as a circumferential sheath or sweet spot of the nerve [40]. The sweet spot of the nerve can be an ideal site for LA deposition as it results in a faster onset with satisfactory block duration. Also, due to its extraneural location, it poses no risk of nerve injury.

Blood Supply to Nerves

Each nerve derives its blood supply from surrounding accompanying blood vessels called the vasa nervorum [41]. Branches from these regional blood vessels enter the epineurium to form a vascular plexus. From the plexus, vessels penetrate the perineurium and enter the endoneurium as arterioles and capillaries. The vascularity of each nerve divides into extrinsic and intrinsic blood vessels. Extrinsic or longitudinal blood vessels lie inside the epineurium and outside the perineurium [42]. Intrinsic or segmental blood capillaries lie inside the endoneurium. The epineurial, or extrinsic, circulation is the critical component of overall neural circulation. Stripping of the epineurium results in a 50% reduction in nerve blood flow and subsequent demyelination of intrinsic nerve fibers [43].

A rich perivascular nerve network (peptidergic, serotoninergic, and adrenergic) around extrinsic blood vessels contributes to the neurogenic control of endoneurial blood flow [44]. As extrinsic blood vessels are under adrenergic control, they are highly responsive to epinephrine-containing solutions [45]. Extrinsic blood vessels are mainly nutritive and communicate with intrinsic blood capillaries inside the endoneurium. The endoneurial capillaries are not anatomically similar to the capillary beds of other tissues. However, physiologically, both serve similar metabolic functions. Atypically larger diameters of endoneurial capillaries and larger intercapillary distances limit their exchange capacity [46]. Similarly, the poorly developed smooth muscle layer of the endoneurial arterioles limits their autoregulation capacity [47]. A higher basal blood flow than the metabolic demands of the nerve provides resistance to neural ischemia. Thus, it protects against nerve dysfunction due to acute ischemia unless blood flow becomes zero.

Nerve physiology (impulse generation and conduction)

An interplay between receptors and stimuli generates the impulse, or AP. Such receptors-stimuli include chemoreceptors - chemicals, photoreceptors - light, baroreceptors - pressure, and proprioceptors - movement. A nerve transmits information through electrochemical impulses (nerve impulses, or APs) that travel from one neuron to another by crossing a synapse. The message is converted from electrical to chemical and then back to electrical [22,48]. Electrical synapses between closely spaced neurons enable the rapid transmission of information. Chemical synapses allow for the release of neurotransmitters from the presynaptic neuron into the synaptic cleft. The released neurotransmitter binds to the postsynaptic neuron, producing an excitatory or inhibitory response [49-51].

**Figure 7:**
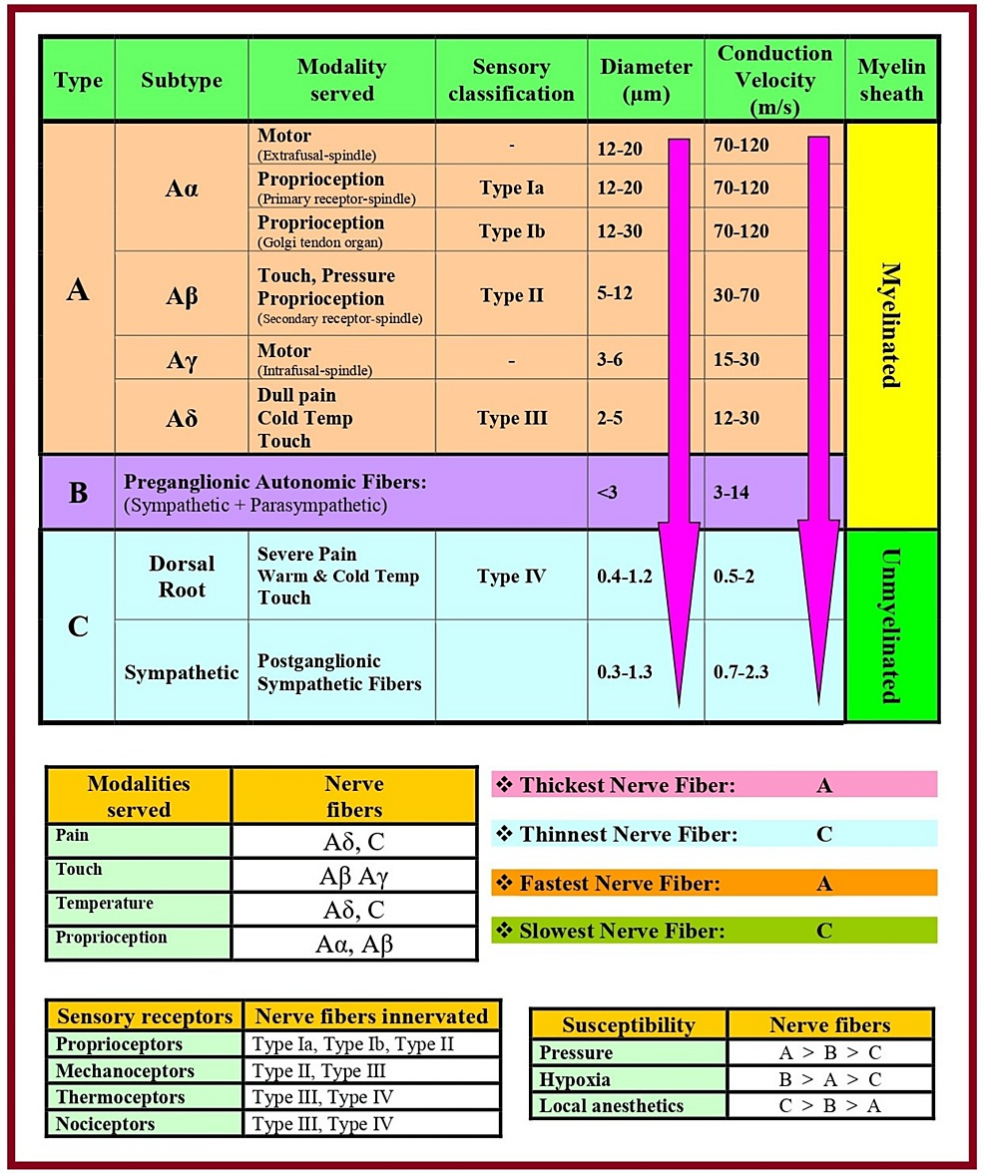
Classification of nerve fibers. Source: This figure was created by the first author (KS) [53,54].

The physiological properties (Table 1) and types of nerve fibers influence the generation and propagation of AP in the nerve [52]. Nerve fibers can be classified based on structure (myelinated/unmyelinated), distribution (somatic/visceral/autonomic), origin (cranial/spinal), functions (sensory [afferent] or motor [efferent]), and neurotransmitter release (adrenergic/cholinergic). Erlanger and Gasser developed the classification system (Figure 7) for peripheral nerve fibers based on axonal conduction velocity, myelination, and fiber size [53,54]. A mixed nerve contains nerve fibers of different types and diameters. The monophasic recording of a mixed nerve impulse is called the compound AP, which is an algebraic summation of the AP of each nerve fiber. The individual nerve fibers conduct such impulses at a speed of up to 120 m/s depending on their types. The change in the membrane potential is due to the movement of ions (sodium, potassium, and chloride) in and out of the nerve cell against concentration and electrical gradients. The resulting AP is driven by ion channel proteins, which are responsible for the transmembrane flux of these ions.

**Table 1:** Physiological properties of the nerve fibers. [52].

Properties on nerve fibers	Description
Excitability and irritability	The nerve fibers are highly excitable structures that respond to several stimuli and can also generate electrical impulses.
Stimulus	Any detectable physical, chemical, or electrical change in the external or internal environment excites the nerve.
	A stimulus must have a minimal intensity, called a threshold stimulus, to be effective.
	The subliminal (weak) stimulus will have no effect.
	The supraliminal (strong) stimulus will produce the same degree of impulse as the threshold stimulus.
Conductivity	The electrical impulses generated in the nerve fibers are propagated along its entire length and to different neurons, muscles, and glands by synaptic connections.
Velocity	The conduction velocity is higher in long and thick myelinated nerves.
	It is higher in homeotherms than in poikilotherms.
Summation effect	Due to the summation effect, many weak (subliminal) stimuli given repeatedly may produce an impulse.
Adaptation or accommodation	Action potential (AP) is produced when a threshold stimulus is applied quickly.
	When threshold stimulus is applied slowly, no AP is produced.
	This phenomenon of adaptation to stimulus is called accommodation.
	Accommodation is the decreased excitability of the nerve fiber due to continuous stimuli.
All or none block	A nerve fiber translates either all of the impulses or none at all.
	Increasing the strength of the stimulus above the threshold level will not affect the AP.
Refractory period	It is the time interval (in milliseconds) during which a nerve fails to respond to a second stimulus, even if it is strong.
	The nerve fibers can conduct one AP at once, i.e., the excitability of the fibers is less during conduction, and hence a new electrical impulse cannot be generated.
Synaptic delay	The impulse takes about 0.3-0.5 milliseconds to cross a synapse, which is necessary for releasing neurotransmitters from the axon terminal and excitation in the dendroid of the next neuron.
Synaptic fatigue	The transmission of nerve impulses across the synapse stops temporarily due to the depletion of the neurotransmitter.
Infatiguability	The nerve fibers are not fatigued upon continuous stimulation.

Ion Channels

**Figure 8:**
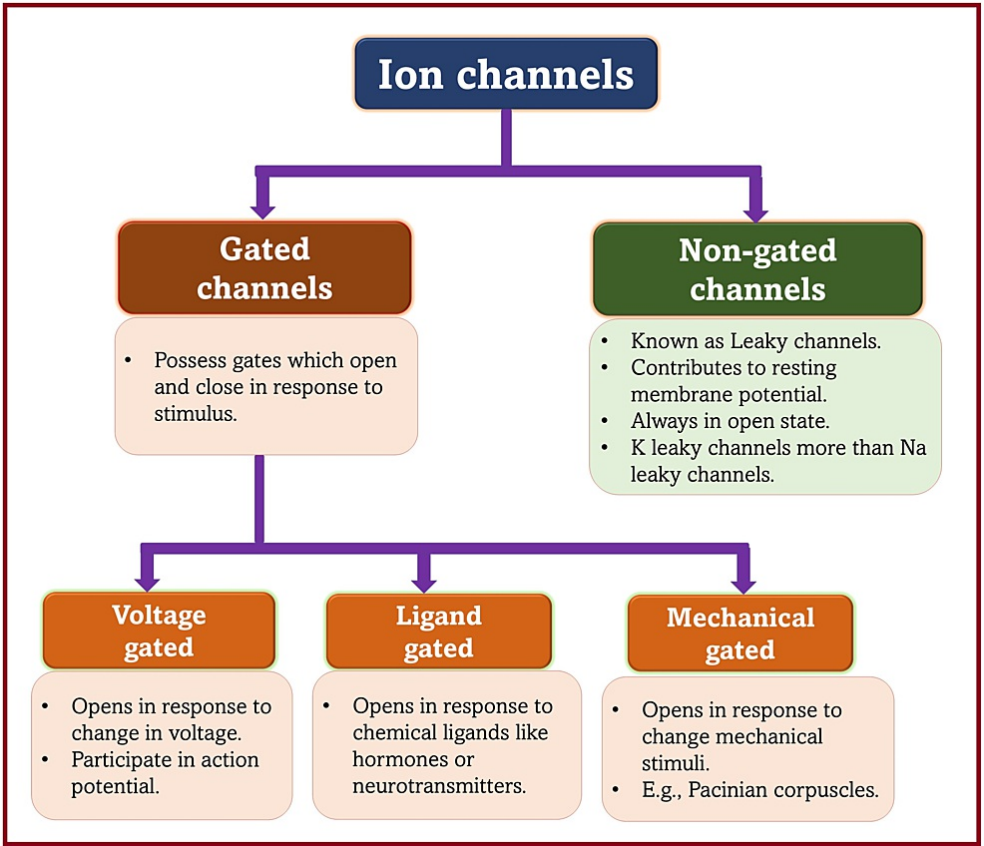
Types of ion channels. Source: This figure was created by the first author (KS) [55,56].

Ion channels (Figure 8) are specialized protein channels on the plasma membrane that play an important role in the excitability of neurons and muscles [55]. They can be gated (to open/close) or non-gated (leaky channels) [56]. Ion channels have selective permeability for certain ions: Na channels for Na ions only and potassium (K) channels for K ions only. Since K channels are more numerous than Na channels, K permeability is greater than Na permeability under resting conditions. Na ions are major extracellular cations, while K ions are major intracellular cations. The movement of ions across the channels always occurs against the concentration gradient, i.e., from higher to lower concentration. For this reason, the movement of Na ions occurs from extracellular to intracellular (Na influx), while the movement of K ions occurs from intracellular to extracellular (K efflux).

Resting Membrane Potential

The resting membrane potential (RMP) is the potential across the cell membrane when the cell is at rest [3]. The non-gated or leaky channels are mainly responsible for RMP [57]. At the RMP, the concentration of K ions is higher intracellularly than that of Na ions extracellularly. It results in a negative charge inside the cell membrane and a positive charge outside the RMP to maintain electrical neutrality [57]. The membrane is said to be polarized when the differences in ion concentration on either side of the membrane give rise to membrane potential. Depolarization is the change in membrane potential in the positive direction upon stimulation. Further positive changes in membrane potential lead to the generation of AP, the final electrical step in integrating synaptic messages at the neuron level [10].

Action Potential

**Figure 9:**
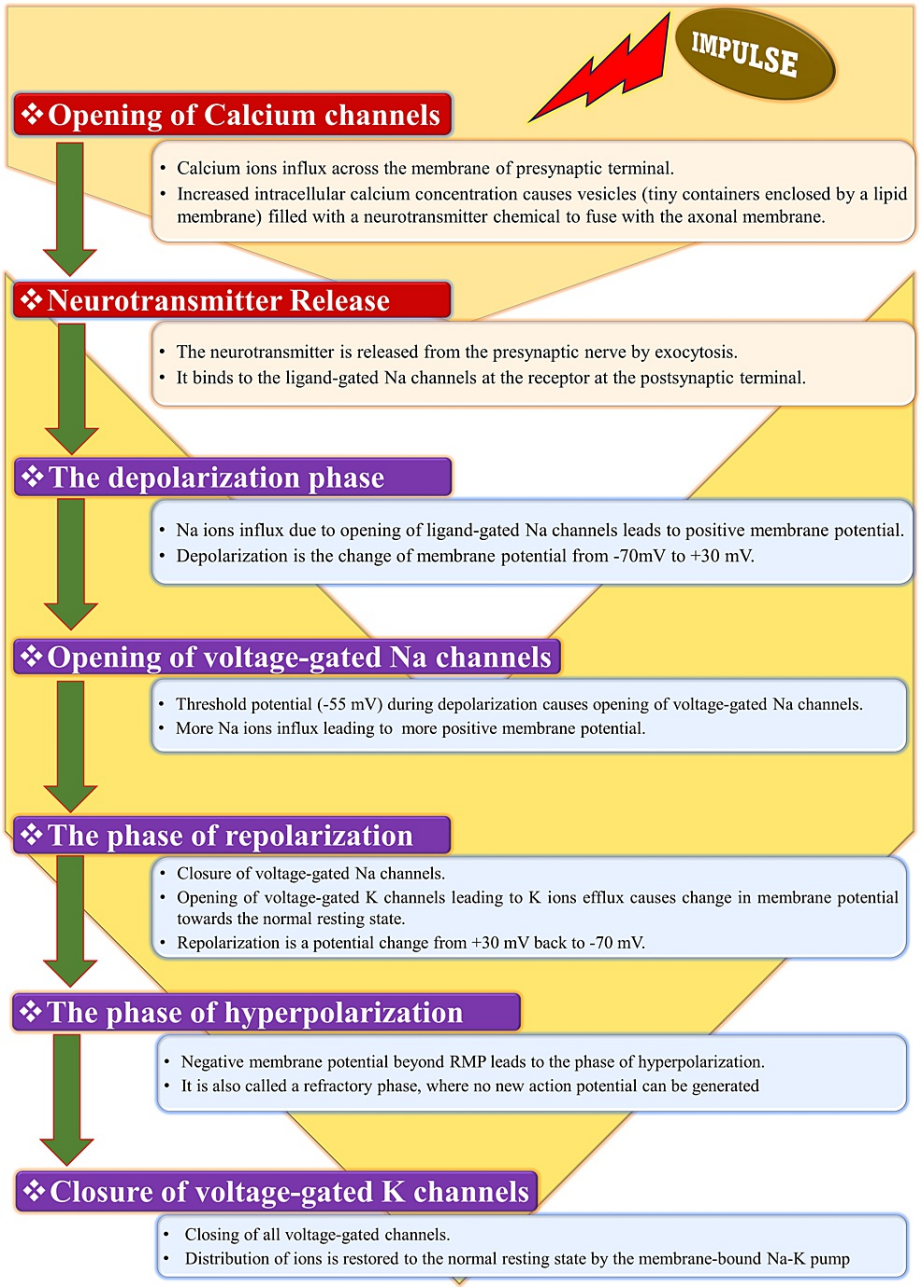
Sequential changes at the presynaptic and postsynaptic terminals during action potential. Na: sodium; K: potassium; RMP: resting membrane potential [3,6,50,51,58]. Source: This figure was created by the first author (KS).

**Figure 10:**
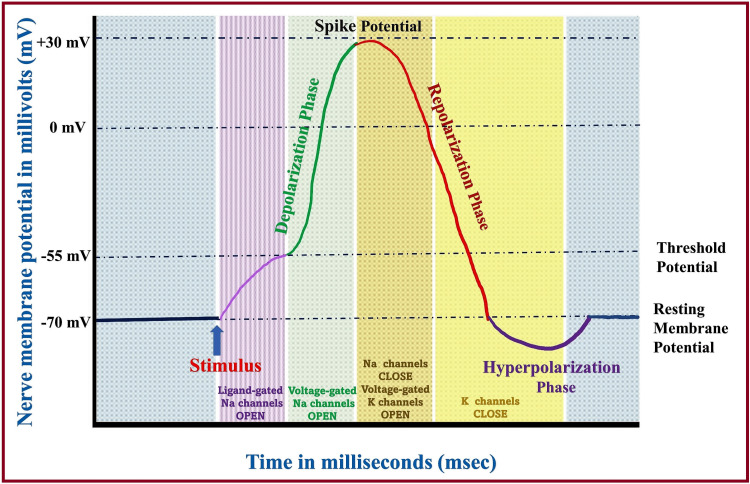
Various phases of the action potential. Na: sodium; K: potassium. Source: This figure was created by the first author (KS).

Each impulse results in sequential changes (Figure 9) in the ion channel proteins from closed to open states depending on the voltage difference across the nerve cell membrane. Such changes mediate various phases of AP (Figure 10) (polarization, depolarization, repolarization, and hyperpolarization) [3,6,50,51,58]. A conducted impulse at the presynaptic terminal activates synaptic transmission by opening calcium (Ca) channels in the axonal membrane. The release of the neurotransmitter into the extracellular space due to the influx of Ca ions leads to a change in the Na distribution across the postsynaptic terminal, resulting in a membrane potential difference. The first stage of the AP is the opening of voltage-gated Na channels, causing more Na to flow inside, leading to more positive membrane potential. The latent phase is the short isoelectric period between the application of the stimulus and the onset of AP [3,6,58]. It is followed by the phase of firing, in which the depolarization initially starts slowly from a minus to a zero potential and then quickly goes beyond zero (overshoot). Depolarization (acceleration) is followed by repolarization (deceleration), which leads to the spike potential. The repolarization phase slowly reaches zero potential and quickly achieves sub-zero (minus) potential. The hyperpolarization phase begins when the repolarization reaches further negative potential below the RMP. In the end, all voltage-gated channels get closed, and the membrane-bound Na-K pump restores the distribution of ions to the normal resting state [3,6]. Nerve AP differs from muscle AP in several ways, as shown in Table 2.

**Table 2:** Comparison between nerve and muscle action potential.

Nerves action potential	Muscles action potential
The resting membrane potential of nerves is around −70 mV.	The resting membrane potential of skeletal and cardiac muscle is closer to −90 mV.
Only the axon is involved during the nerve action potential.	The entire muscle cell membrane is involved during the muscle action potential.
Duration: 0.5–2 milliseconds.	Duration: 1–5 milliseconds (skeletal muscles) and 10–300 milliseconds (cardiac and smooth muscles).
Nerve conduction velocity is 18 times faster (in the fastest nerve fiber) than muscle.	Slower conduction velocity.

Refractory Period

The period immediately following stimulation, in which the excited nerve remains unresponsive to further stimuli, is called the refractory period [3,59]. Due to this, a second AP cannot be generated during the firing phase of the nerve. The refractory period facilitates the unidirectional propagation of the AP along the axon. It is further divided into absolute and relative refractory periods. The AP is due to the opening of voltage-gated Na channels, which get inactivated after spike potential. They cannot be reactivated immediately, as recovery from the inactivation is time- and voltage-dependent. The period (about 1-2 msec) from the initiation of the AP to just after the spike potential is called the absolute refractory period (ARP) [3,59]. Therefore, ARP includes the depolarization and two-thirds of the repolarization phases. No further AP can be generated during ARP, despite the stronger or suprathreshold stimuli. The K channel opening after inactivating the Na channel results in K ion efflux. Subsequent recovery of the Na channel after inactivation allows for a second AP generation [3,9]. However, due to sustained K efflux, there is a tendency to counteract further depolarization. Therefore, a stronger (than normal) stimulus is required to generate a second AP. This period (about 3-4 msec) after the ARP that allows the firing of a second AP (with stronger stimuli) due to Na channel recovery is called the relative refractory period [3].

Factors influencing the impulse conduction

As mentioned before, impulse propagation depends on the physiological properties of nerve fibers (Table 2) [52]. The loss of amplitude during the impulse propagation along the nerve fiber determines the conduction speed, which further depends on the presence of myelin. The presence of myelin greatly increases conduction velocity by decreasing the capacitance (ability to store charge), spreading more charge along the rest of the axon, and increasing the depolarization rate and propagation speed [60]. Also, the tightly wrapped myelin around the axon increases the transmembrane resistance of ion flow. Therefore, the APs develop only at the myelin gaps or nodes of Ranvier. The voltage of adjacent areas is affected by the opening of the voltage-gated Na channels, which are much denser at nodes of Ranvier and sparse to absent under the myelin sheath.

Fast Versus Slow Conduction

The conduction speed in myelinated nerve fibers is 50 times greater than that of unmyelinated nerve fibers. In myelinated nerve fibers, the depolarization occurs only at the nodes of Ranvier, the unmyelinated portions of the myelinated nerve fibers that contain a high density of voltage-gated Na ion channels. The current carried by ions flows from node to node through extracellular fluid. The electrical currents generated at each node of Ranvier are conducted with little attenuation to the next node in line, where they remain strong enough to generate another AP. Therefore, APs in a myelinated axon effectively "jump" from node to node, bypassing the myelinated stretches in between [61,62]. It results in a faster propagation speed than even the fastest unmyelinated axon can sustain. Such conduction in myelinated nerve fibers is known as saltatory or nonhomogenous conduction [61,62].

In unmyelinated nerve fibers, the impulse propagates across the surface of the axolemma, causing Na ions to flow into the cell during depolarization. The opening of adjacent voltage-gated ion channels allows more Na ions to enter, leading to further depolarization along the length of the axon. It is a step-by-step depolarization of each portion of the length of the axolemma [61,63]. The propagation speed depends on the rate of depolarization of the axon segment in front of the AP. It is influenced by the concentration of the Na channels and the diameter of the axon. Such conduction in unmyelinated nerve fibers is known as continuous or homogenous conduction [61,63].

The Margin of Safety for Impulse Conduction

During impulse conduction, the current flowing through the nerve is five to ten times the current required to depolarize it. It is known as the margin of safety for impulse conduction [64,65]. Some diseases or drugs can decrease membrane excitability by decreasing the amplitude of AP, conduction velocity, and depolarization rate. It is known as decremental impulse conduction [64,65]. However, conduction failure cannot occur until it exceeds the margin of safety. LA blocks nerve conduction by blocking the Na channels [64,65]. For that, 80% of Na channels must be blocked to exceed the margin of safety and achieve a conduction blockade [64,65]. It is believed that Na channels must be blocked in at least three consecutive nodes of Ranvier for axonal conduction blockade [64,65]. For an effective conduction block, the distance between two nodes of Ranvier must be known to deposit LA around the nerve at a minimum distance covering at least three consecutive nodes of Ranvier. Each node of Ranvier can span around 1-2 µm, and the internodal distance can be up to 2 mm long, containing 5 mm of myelin spiral [66,67].

Nerve Fibers Arrangement

**Figure 11:**
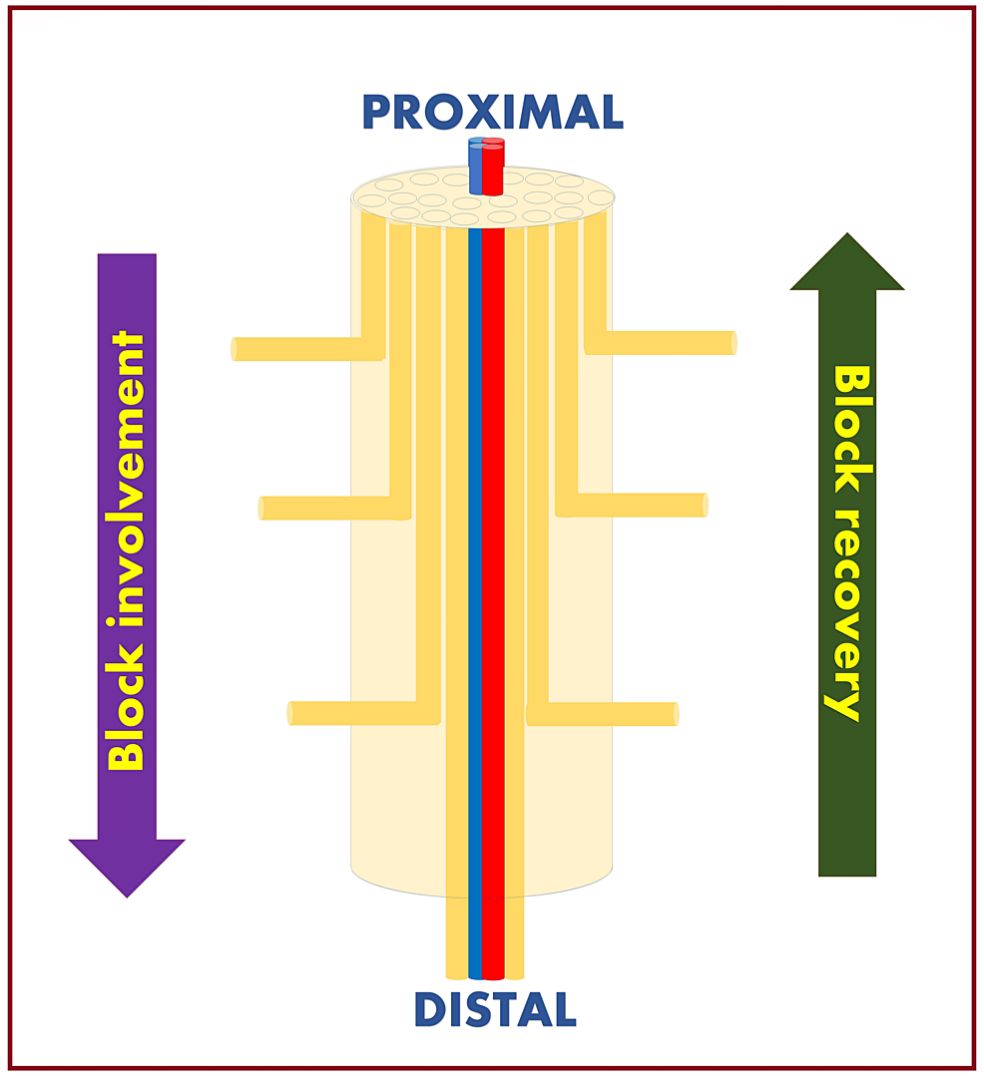
Mantle and core arrangement of the nerve fibers in the peripheral nerve. Source: This figure was created by the first author (KS).

In the peripheral nerve, the mantle (outer) fibers innervate the proximal structures, and the core (inner) fibers innervate the distal structures (Figure 11). Such an arrangement causes anesthesia/analgesia to progress in a proximal-to-distal direction. The core fibers are highly vascular, so the recovery occurs in a distal-to-proximal direction [68,69].

**Figure 12:**
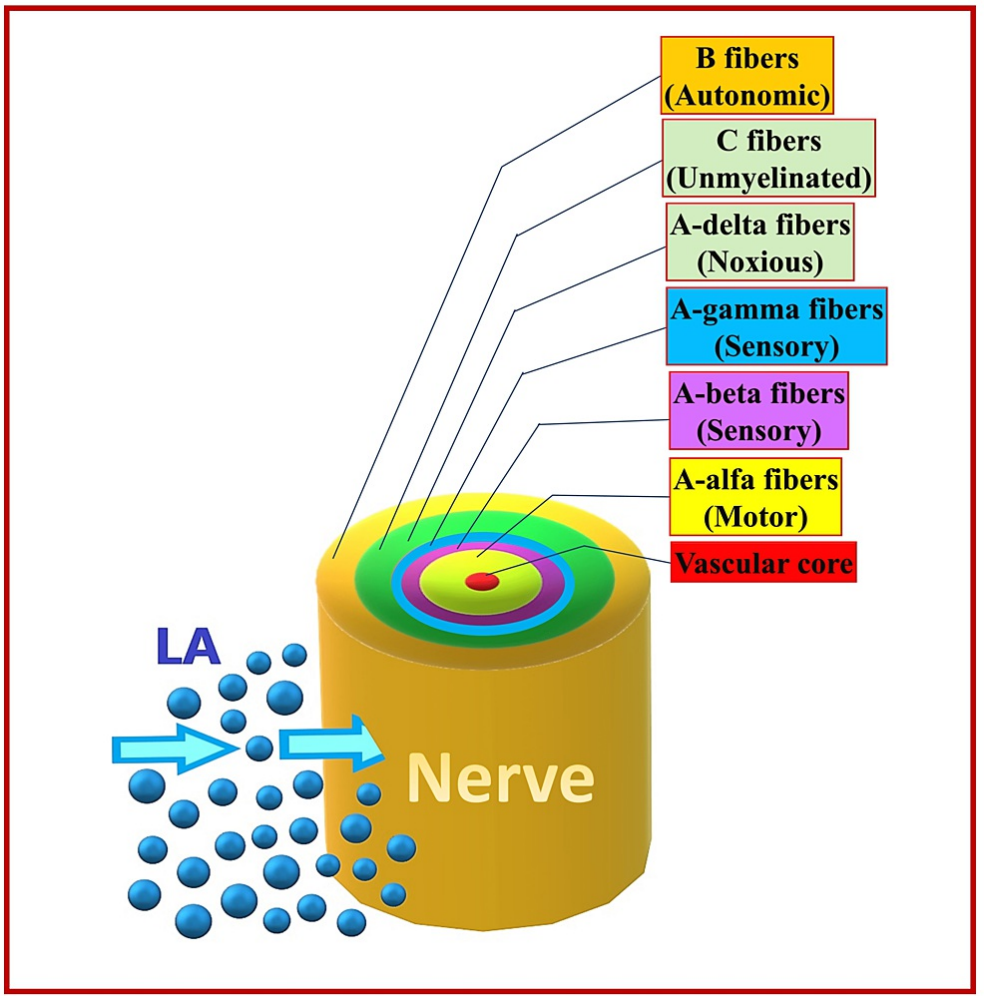
Location of various nerve fiber types in the peripheral nerve. LA: local anesthetic. Source: This figure was created by the first author (KS).

Differential block by the LA can be due to the similar arrangement of various types of nerve fibers in an individual nerve (Figure 12). Mantle fibers mainly include (from outside to inside) B-fibers (autonomic), C-fibres (unmyelinated), Aδ (noxious), and Aβ fibers (sensory). The core fibers mainly include Aα fibers (motor) [70]. Depending on the fiber type and size, the sequence of block onset is B fibers> C fibers = Aδ fibers> Aγ fibers> Aβ fibers> Aα fibers. Therefore, sympathetic block occurs before sensory block, which in turn occurs before motor block. Block reversal occurs in a reverse manner (i.e., Aα > Aβ > Aγ > Aδ = C > B) [71]. Therefore, the motor block lasts the shortest, while the autonomic block lasts the longest. The diffusion of LA from the mantle to the core fibers depends on the concentration gradient. For this reason, a high LA concentration is required to involve motor fibers (core fibers). In contrast, a low LA concentration can only involve mantle fibers (sensory fibers), allowing for a motor-sparing effect [68,69].

## Conclusions

In summary, detailed knowledge of the flexiform structure of the nerve allows for a more logical and safer approach to avoiding dreaded complications like nerve/plexus damage and associated litigation. Knowing such intricate details about the target structure, the nerve, can help in many aspects: in planning and safely administering the block, in choosing the right LA ​​dose/concentration and safe placement sites, and in understanding the block pattern to assess it further. It also helps achieve some procedure-specific goals, like providing a selective sensory block for postoperative analgesia, an anesthetic block for surgical procedures, and patient-centered, safe RA. We believe updating this fundamental knowledge should be an important aspect of RA practice and training.
